# New application to remediate drinkable groundwater from excess of hardness ions by using sodalite bearing modified illite

**DOI:** 10.1007/s10653-022-01340-7

**Published:** 2022-08-08

**Authors:** Atef Mohamed Gad Mohamed, Al-Shimaa Roshdy Mohamed Ali, Abd El Hay Ali Farrag, Mahmoud Mohamed Ahmed Mohamed

**Affiliations:** 1 Assiut and New Valley Company for Water and Wastewater, Asyut, Egypt; 2 Geology Department, Faculty of Science, Assiut University, Asyut, Box. No. 71516, Egypt; 3 Department of Chemistry, Faculty of Science, New Valley University, Kharga, 72511 Egypt

**Keywords:** Groundwater, Illite, Exothermic, Hardness ions, Alkaline modification, Sodalite, XRD, XRF, SEM

## Abstract

Calcium Hardness (Ca. H) and total Hardness ions in drinkable groundwater cause great problems for the entire world especially, the population communities which are located far from surface water sources. The present study investigates the use of Sodalite Bearing Modified Illite (SBMI) as a sustainable and new technique to eliminate these ions from drinkable groundwater to compatible with the instruction of the World Health Organization. The methodology was achieved by using a new method to remove these ions’ excess calcium Hardness and total Hardness depending on two main processes; the adsorption as a first step and the coagulation-flocculation-sedimentation process as a second step. The results of this study were achieved through conducting three tasks; (1) Chemical analysis surveys for all the groundwater wells, to determine the areas which are more affected by these salts, and plot them on the location maps. (2) Conducting the alkaline modification of the Illite ore to obtain the (SBMI) which has a high surface area and high adsorption ability, and it had been characterized by using XRD, XRF, SEM, and FTIR techniques. (3) The experimental studies were conducted to evaluate the effect of the modified Illite on raw groundwater containing a high concentration of hardness ions, through the batch studies to determine the factors which affected its ability for removing these ions from groundwater. The present study illustrated that the removing efficiency for both total hardness (Ca. H + Mg. H) and calcium hardness (Ca. H) reached about 98%. Finally, the present study recommended using this technique, when there is a requirement for large quantities of treated water at a low cost.

## Introduction

In Egypt, the Nile River is considered the main source for drinking and agricultural purposes for most Egyptian populations. So they are lives around its two banks. About 90% of Egypt’s area is arid desert due to its climate which is characterized by poor rainfall. Thereby the groundwater is considered the only source of fresh water for these localities away from Nile valley. But mostly this groundwater contains high ratios of the calcium and total hardness (Ca + Mg) ions over permissible limits of WHO (2006). This is due to two factors; (1) Climate effect; the climate change has a significant impact on the hydrological cycle and water resources. The temperature rises caused by climate change alter precipitation patterns in various parts of the world (Barebari et al., 2021). The hydrological cycle and water availability are affected by changes in temperature and precipitation. Climate change has also had an impact on groundwater levels in aquifers due to recharge changes (Barbieri et al., 2021; El-Rawy et al., 2021). So with the increase in this global warmth, the evaporation rate increases leading to a decrease in groundwater recharge, and the concentration of Ca, and Mg ions is increased. The climate of Assiut Governorate (study area) is characterized by long and hot summer, short cold winter (December to February periods), low rainfall, and high evaporation (Megahed et al., 2019). (2) The nature of the bearing bed of groundwater; the quaternary aquifer is considered the essential aquifer bearing the groundwater in Egypt, but usually, this groundwater was affected by the rock contents of the aquifer and its other surrounding environments. In the present study, groundwater flows through aquifer beds from the upper parts in the south (thickness of beds about 200 m) to the lower parts in the north direction (thickness of beds about 300 m) along the study area (Megahed et al., 2019). So the hardness ions can occur in groundwater by the dissolution of limestone, magnesium limestone, gypsum, anhydrite, calcium-bearing minerals, and other minerals at higher concentrations as geogenic sources, or due to the anthropogenic activities (Barbieri et al., 2019; Dehnavi et al., 2011; Ricolfi et al., 2020). like industrial effluents such as the chemical and mining industry or the excessive use of lime in soil and the agriculture field (Abdelhafeez et al., 2022; Abdel-Raheem et al., 2022; Tolba et al., 2022; Flanigen et al., 2010). Many problems may occur by an excess of hardness ions in the drinkable groundwater; (1) physically like bad taste and odor, (2) chemically like permanent hardness which making scales in pipelines, causing many technical problems (Farrag et al., 2017; Fathy et al., 2019).

According to the Egyptian Standard No. 458/2007, the total and calcium ions concentrations must not exceed the permissible limit (500 mg/L and 350 mg/L, respectively). So many conventional treatment methods are used to reduce these undesirable ions (Widodo et al., 2020; Abd Aziz et al. 2019; Cabiguen Jr et al. 2018; Vosoogh et al., 2017) (1) Reverses Osmosis (RO) is the most common technique used to eliminate these hardness ions from raw water. But its high cost and low capacity for producer of treated water. (2) Ion exchange is the advanced technology to produce a relatively higher quantity of water than produced by RO. but it needs a lot of regeneration intervals to reactivate. Sodalite is a zeolite with a six-membered ring aperture framework structure (Jiang et al., 2012). Sodalite has garnered a lot of attention because of its small pore size and high adsorption capacity (Arieli et al., 2004), waste management (Buhl et al., 2003), hydrogen storage (Buhl et al., 2005), hydrogen separation (Julbe et al., 2003), and catalyst support (Ogura et al., 2008). SO, zeolite, also known as molecular sieves, is a porous material with a three-dimensional framework structure of crystalline aluminosilicates that creates consistently sized pores. Al_2_O_3_·*y*SiO_2_·*w*H_2_O is the empirical formula for zeolite. Where *y* is 2–200 and *w* is the water contained in the intra crystalline channel of zeolite (Flanigen et al., 2010), the frameworks are made up of tetrahedral [SiO_4_]_4_ and [AlO_4_]_5_ that are corner-shared to generate various open configurations. The present study intended to prepare modified Illite to obtain Sodalite Bearing Modified Illite (SBMI) and use it as a novel application for removing hardness ions from raw drinkable groundwater in combination with Alum. Thereby the SBMI was used to adsorb hardness ions dissolved in raw water as the first step followed by the second step for removing the SBMI containing the hardness ions as suspended solid particles by alum via coagulation-flocculation- sedimentation process then followed by sand filtration. The essential factors which affect the removal efficiency like pH, appropriate dose, and others are studied.

## Materials and method

### Materials

- Groundwater samples were taken from about 235 sites producing drinking water covering all localities of Assiut governorate to identify the most contaminated of them by total and calcium hardness. All well samples were taken monthly during the period 2020–2021 for the analysis, the average of these data were plotted in GIS maps.
- The majority of these wells penetrate the quaternary aquifer with the depth ranging from 70 to 100 m, and only a few productive wells in depths less than 70 m. At the borders of the east and west sides near the scarp of the limestone plateau, there are some wells more than 150 m in depth.
- EL-Bora groundwater used for all the parts of study. Only in the part of fully capacity of 7 g/l of SBMI for adsorption using three different groundwater concentrations of total and calcium hardness (Nagaa Sabaa, EL-Bora, Refa)
- Natural Illite obtained from Abu-Tartour area, New Valley region, Egypt, away from study area (Assiut governorate) by about 250 km, located between Longitude 30° 5′ 7″ east and latitude 25° 22′ 41″ North, to prepare Sodalite bearing modified illite (SBMI).
- Sodium hydroxide (NaOH with purity 98%).
- Commercial Aluminum Sulfate (Al_2_ (SO_4_)_3_·18H_2_O).
- Milli-Q water.

### Methods

#### Modification of Illite ore

##### Physical modification

The natural Illite ore must be crushed by using an agate mortar and sieved with a mechanical sieve to get grain sizes less than 0.3 mm.

##### Chemical modification

The chemical modification of the Illite ore is conducted by mixing it with NaOH pellets in ratio 10:1 solid to solid, and mixed them with milli Q water. The prepared paste exposed to sunlight for one week. The Sodalite Bearing Modified Illite (SBMI) was resulted from the transformation of elements depending upon exothermic temperature reaction reached to 50 °C without any calcination process (direct method). The silica-alumina ratio in the prepared SBMI was 2.7, this condition of reaction fall within the range required for sodalite formation as mentioned by (Deng et al., 2006; Rabiller et al., 2004).

##### Preparation of alum solution

For the preparation of stock alum solution 1%, it was using commercial Aluminum Sulfate (Al_2_ (SO_4_)_3_·18H_2_O) with Milli-Q water according to the American Public Health Association (APHA, 2015) to achieve the coagulation-flocculation-sedimentation process.

#### Optimization study

In order to conduct the experiments of the optimization study for the different adsorption parameters like adsorbent dose, contact time, and the required dose of alum to purify raw groundwater from SBMI containing hardness ions and other impurities after adding the adsorbent materials (SBMI);

1. Using the Jar test instrument having beakers with a different capacity 1000–3000 ml.
2. The experimental treatment studies were conducted by using the natural groundwater well of EL-Bora Village as a source of a constant initial concentration of total hardness 680 mg/L and calcium ions 580 mg/L, and using the Jar test machine. The batch experiments are performed at room temperature 25 °C for the elimination of these ions from raw groundwater.
  In this study, the adsorption process for the hardness ions by SBMI as a first step will take 25–30 min at 100 rpm (Pramesti et al., 2021), and then the alum dose is added in the same mixing rate for 2 min to achieve the coagulation process and then flocculation process which takes 30 min at 25 rpm, and finally the sedimentation process takes half an hour, and then the filtration process takes place with a filter paper (110 mm). The concentration of hardness parameters Total Hardness (T. H), Calcium Hardness (Ca.·H) were determined by using the titration method (Standard Methods for the Examination of Water and Wastewater 23 RD (2340 C. EDTA Titrimetric Method). The concentration of magnesium hardness (Mg. H) calculated by subtracting the concentration of T.H from Ca. H (T. H–Ca. H). The quality control for total hardness are (LOD = 1.003, LOQ = 3.343) and calcium hardness are (LOD = 1.188, LOQ = 3.962).
3. The removal efficiency of SBMI was calculated by applying the following equation (Eq. 1).
  1 $${\text{Removal}}\,{\text{ efficiency }}\left( \% \right) \, = \, C_{0} {-} \, C_{e} /C_{0} \times 100$$
  where *C*_0_ and Ce are the concentrations of hardness ions in before and after treatment (mg/l), respectively.

#### Scenario about the recommended water treatment

**Fig. 1:**
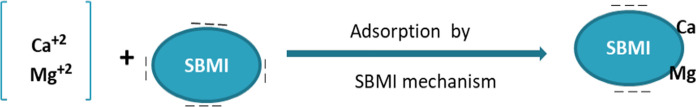
Schematic diagram to illustrate the adsorption mechanism concept for Mg and Ca ions by SBMI (first step)

The mechanism of adsorption by SBMI depended on its framework electronegativity which resulted from the substitution of some SiO_4_ tetrahedrons by [AlO_4_]^−1^ which can be balanced by the cations such as Mg^+2^, Ca^+2^ dissolved in raw groundwater, and this concept is in agreement with Kusumastuti et al., (2018). It can be draw this adsorption mechanism concept of the hardness ions by SBMI as in (Fig. 1) as a first step.

**Fig. 2:**
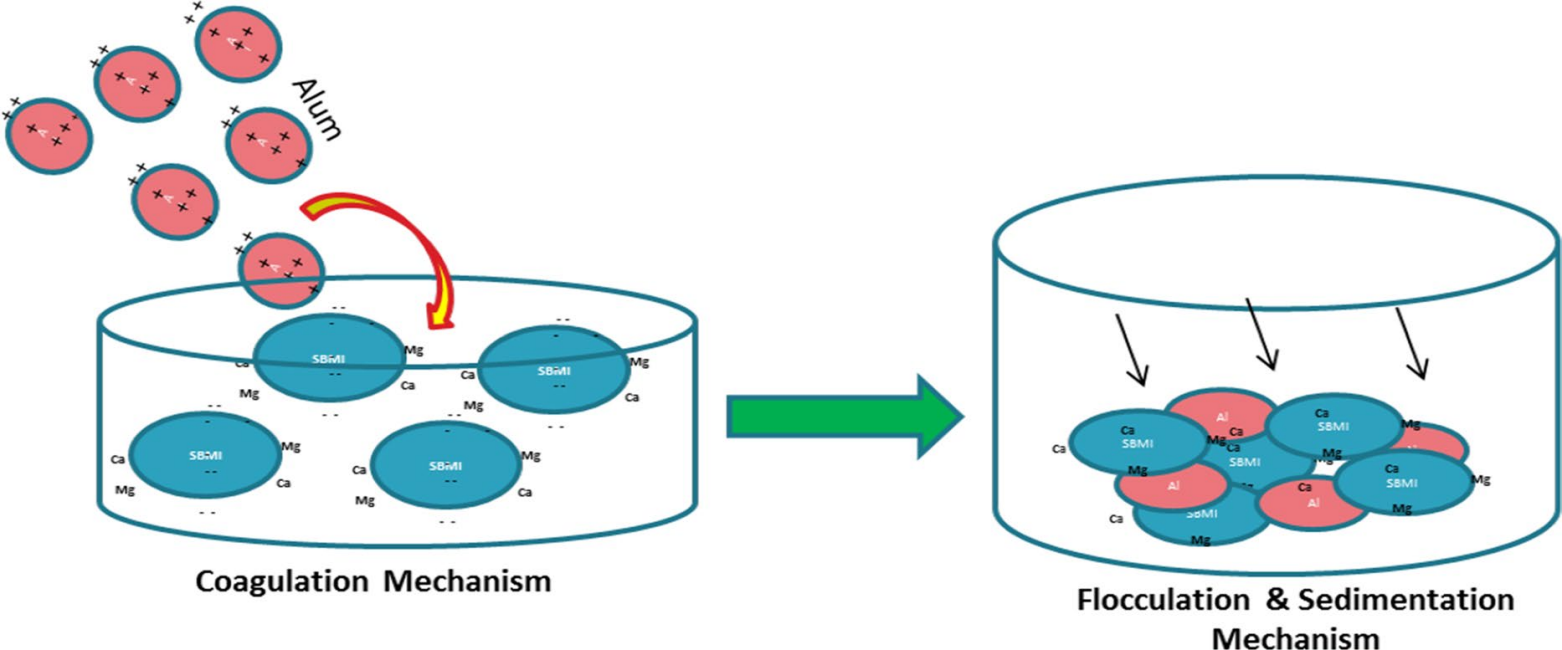
Schematic diagram to illustrate the settling of Ca and Mg ions bearing SBMI particles and other impurities as a turbidity by alum (second step)

**Fig. 3:**
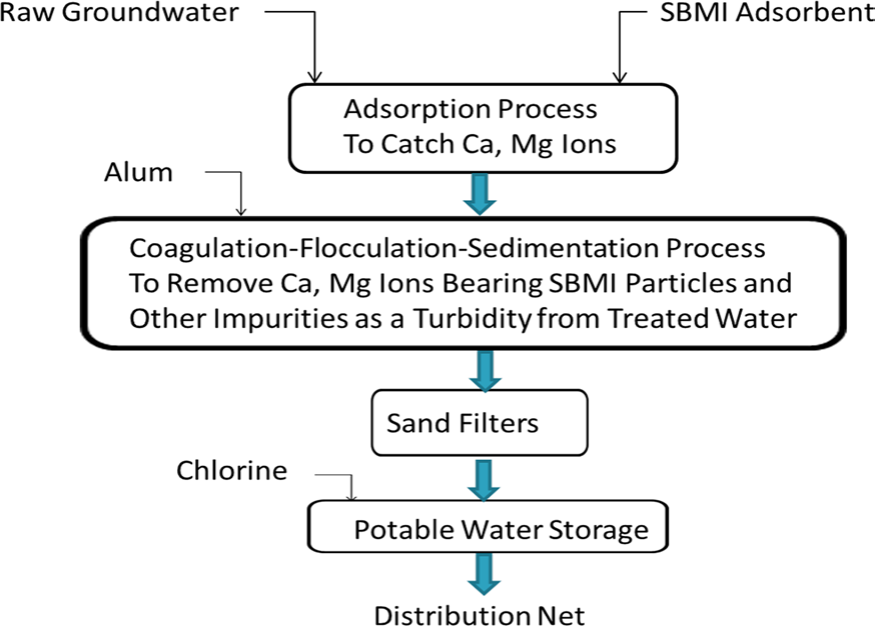
Schematic to illustrate the recommended water treatment Scenario by using SBMI and alum

The second step was comprised of using the alum solution for achieving the Coagulation- flocculation and Sedimentation mechanisms to eliminate the SBMI particles containing hardness ions and other impurities suspended particles in form of turbidity in drinkable groundwater as shown in (Fig. 2). Finally, (Fig. 3) shows the recommended water treatment concept by using SBMI and alum.

#### Characterization techniques for the raw Illite and (SBMI)

In this work the instrumental techniques which used for characterizing the raw and modified illite were (X-ray fluorescence) XRF Philips PW 1480, X-ray diffractometer Shimadzu XRD-6000, (Scanning electron microscope) SEM JEOL JSM-6360LA, (Fourier transform infrared) FTIR spectrophotometer shimadzu prestige 21.

## Results and discussion

### Assiut groundwater quality status (Chemical analysis survey)

**Fig. 4:**
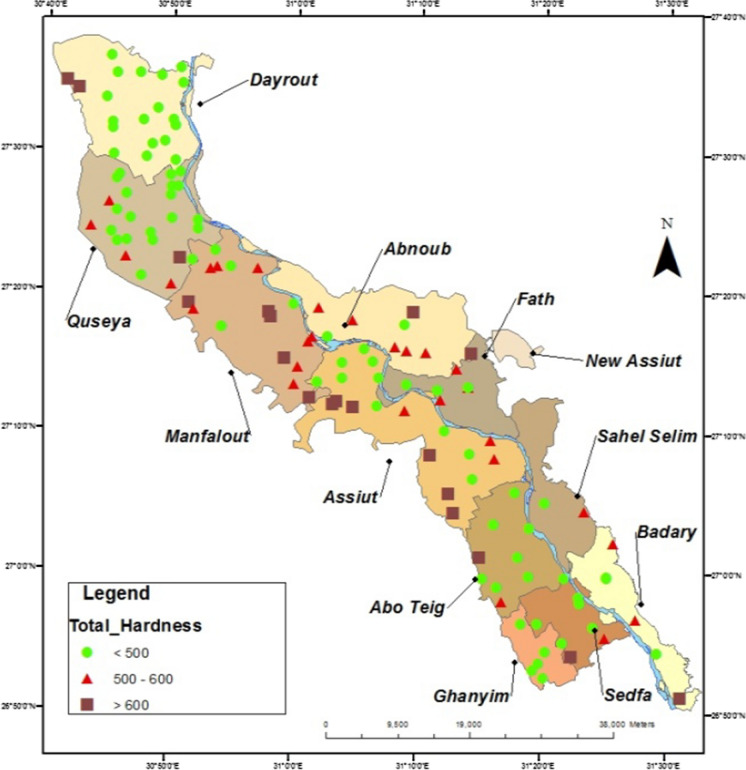
The Total Hardness Distribution (Concentrations and Sites) in Location Map of Assiut Groundwater

**Fig. 5:**
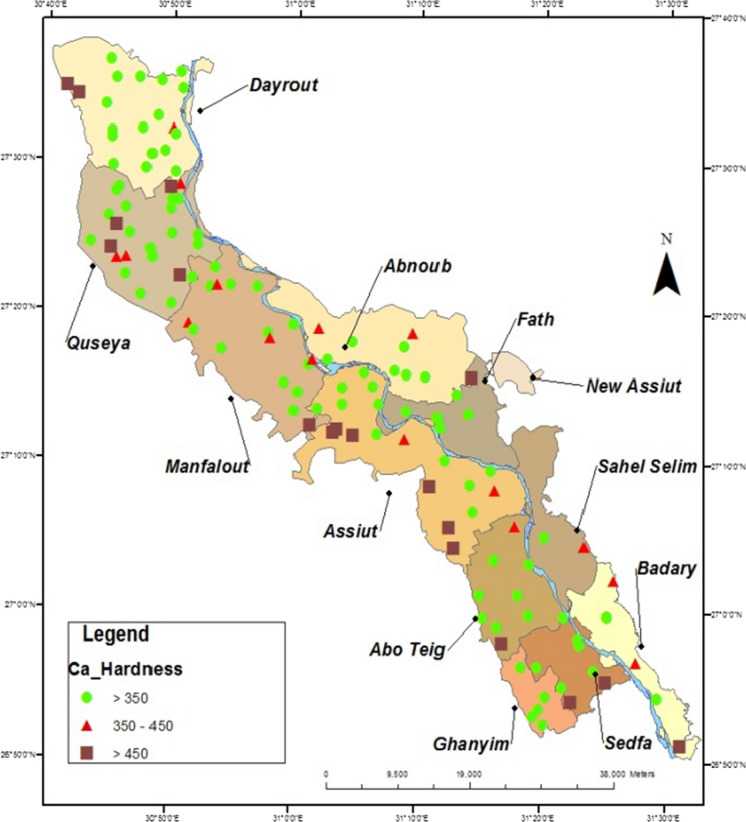
The Ca Hardness Distribution (Concentrations and Sites) in Location Map of Assiut Groundwater

Datta and Tyagi (1996) Interpreted the occurrence of Ca and Mg ions in groundwater were resulting from the dissolution of CaCO_3_, and Ca, Mg (CO_3_)_2_ presented in the limestone and dolomite rocks by the action of groundwater that flows through it, that is responsible for increasing the concentrations of calcium and magnesium ions in groundwater. Also, (Dehnavi et al., 2011) reported that water containing CO_2_ or carbonic acid can easily dissolve the carbonate minerals available in its flow path, and this process is known as a carbonate weathering or ion exchange. In this paper, the results of analyzed groundwater samples collected from all wells representing all villages of Assiut governorate were classified according to their total and calcium hardness contents. Maps were then plotted to locate wells and also the concentration of total and calcium hardness. In Assiut governorate, more than 850 productive wells are owned by Assiut & New Valley Company for drinking water and wastewater. The results of these good samples were compared to the permissible limits of WHO and the Egyptian Standard No. 458/2007. By monitoring the averages of these results, it has been subdivided each parameter of these results into three groups according to its concentration in the groundwater and then plotted on a separate map to illustrated its distribution along the study area by using GIS techniques. It can be isolated about 152 sites were over these permissible limits in total and calcium hardness parameters are sited in location maps as in (Figs. 4, 5). The percentage of non-identical samples according to their total and calcium hardness is in the range of 35% of the examined groundwater samples. The studied area is classified based on the degree of total hardness to three ranges. The first range contains concentrations less than 500 mg/L, where includes about 30% of the total non-identical samples. The second range has total hardness between 500 and 600 mg/L the category represents about 60% of the non-identical samples. The third range has a total hardness higher than 600 mg/L. it includes about 10% of the non-identical samples as in (Fig. 4). In the studied area the behavior of calcium hardness is not significantly different from total hardness, the first range, containing calcium hardness less than 350 mg/L, represents about 30% of the non-identical samples, and the second range, contains calcium hardness between 350 and 450 mg/L, it represents about 60% of the non-identical samples, and the third range, having calcium hardness higher than 450 mg/L, it represents about 10% of the non-identical samples (Fig. 5). The maps in Fig. 4, 5 show that the concentrations of calcium and total hardness ions are increased away from the Nile River due to the weakness of recharge of groundwater by surface water. These results are in agreement with the results studied by Megahid et al. (2019) which mention that near the Nile River and other surface water, the groundwater quality is greater, in opposite low groundwater quality was observed at the Nile Valley’s border, possibly as a result of the nearby Limestone plateau’s influence.

### Characterization studies

#### XRF analysis

The chemical composition analysis occurred by XRF which illustrate that SBMI contained silica SiO_2_ and alumina Al_2_O_3_ as a major compound and other metal oxides, such as Fe_2_O_3_, CaO, MgO, Na_2_O, and K_2_O as a minor compound show them in (Table 1).

**Table 1:** The XRF analysis for Illite ore and modified Illite

Parameter (Mass %)	Na_2_O	MgO	Al_2_O_3_	SiO_2_	SO_3_	K_2_O	CaO	MnO	Fe_2_O_3_	Si /Al Ratio
Natural Illite	0.19	2.87	*19.7*	*52.5*	0.84	4.88	1.12	0.035	8.28	*2.66*
Modified Illite	4.96	2.54	*17.3*	*46.5*	1.16	4.29	1.03	0.049	7.53	*2.68*

#### X-ray diffraction (XRD) analysis

**Fig. 6:**
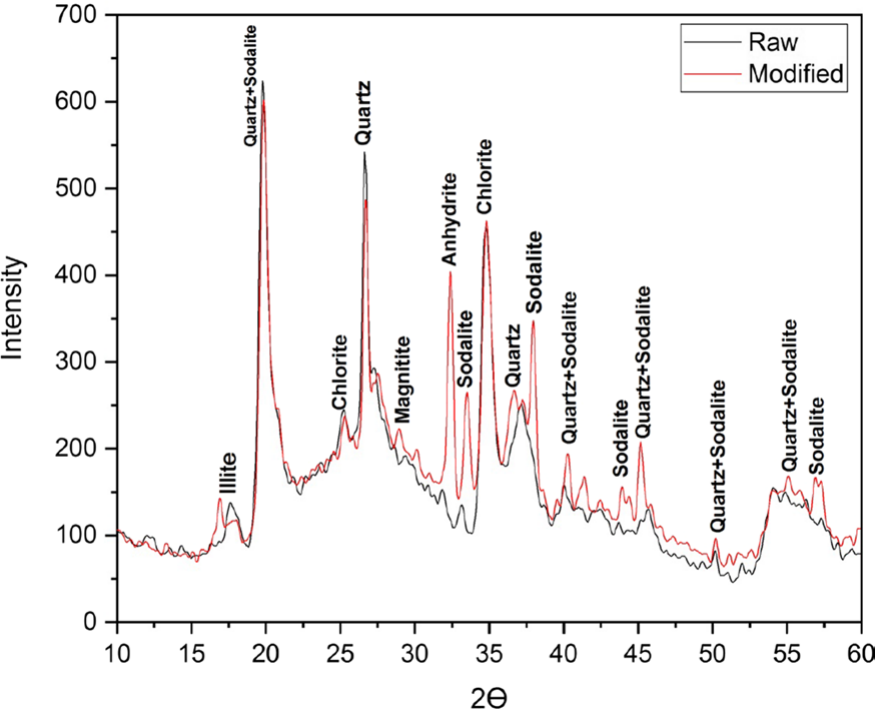
XRD pattern of raw illite and modified illite with new peaks of sodalite crystals formation

The changes in structure which occurred in the clay ore due to the alkaline treatment are studied using X-ray diffraction technique. Figure 6) shows the XRD profiles of the untreated and alkaline treated Illite. The intensity of the XRD peaks of Illite increases on alkaline treatment and its inner structure may be affected. The X-ray diffraction pattern of Sodalite displays the characteristic peak at 2θ values (26.72°, 34.03°, 38.00°, 43.46°, 51.92°, 54.86°) that agree with Rabiller et al., (2004) as show in (Fig. 6).

#### Scanning electron microscope (SEM)

**Fig. 7:**
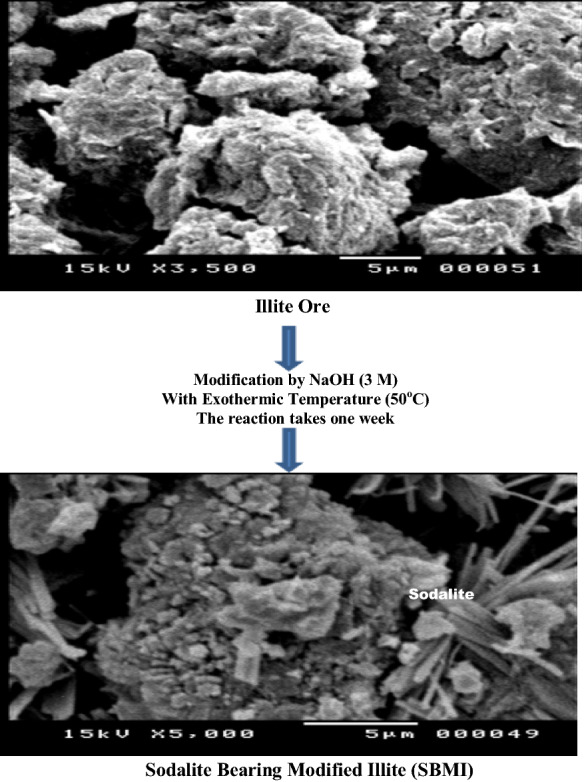
SEM Images were illustrated the morphology of Illite Ore crystals at the upper image before modification, and morphology of the modified illite crystals with synthetic phase of sodalite crystals at the below image

The morphological structure of the Illite ore and its modification samples are studied by using scanning electron microscope SEM. The SEM micrograph of natural clay in (Fig. 7) reveals of hexagonal crystals with sharp edges, rough surfaces. While the SEM micrograph for the modified Illite reveals the presence of elongated crystals that appeared from the reaction between Illite and sodium hydroxide to form Sodalite (Belever et al., 2002). This result in agreement with John Walther (1996) who proved that when aluminosilicates treated or placed in an alkaline medium, they would hydrolyze and condense to form a new structure.

#### FTIR spectra analysis

**Fig. 8:**
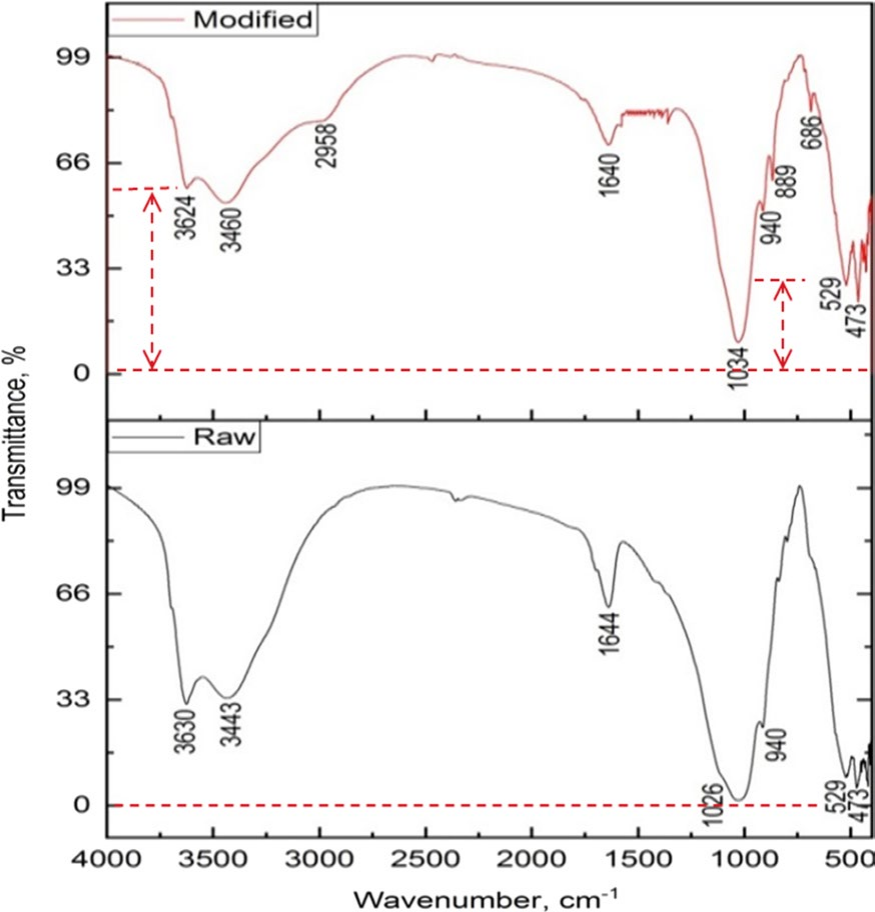
FTIR spectra were illustrated a compasison between the tenisty of transmittance through Illite ore at the upper image before modification, and tenisty of transmittance for Sodalite bearing Modified Illite SBMI after modification process

In this section of FTIR spectra, interpretation was focused on the transmittance parameters to characterize the modification influence on raw Illite, Therefore, the transmittance is directly proportional with the porosity degree of the materials which the light passes through it, the transmittance may be regarded as a porosity index of the sample, this in agreement with the reported that by Sun et al. (2005) the porosity material tends to have a high transmittance value. it is found that the transmittance light of the sample after alkaline modification increased than before the modification process as seen in peaks of band 3624 cm^−1^ and 3460 cm^−1^, which are characterized for the founding of hydroxyl groups and also confirms the formation of Sodalite phase which has high porosity had to lead to increasing in transmittance, and these results were agreement with the mentioned obviously by Sperberga et al., (2011). The formation of the Na–O–Si bond frequency is a little bit lower in the SBMI matric than in untreated Illite signifies that changes have taken place in the bonding structure within individual tetrahedra. The possible change in the coordination number of Al from six to four could cause small peaks to appear at approximately 889 cm^−1^, and 686 cm^−1^ for SBMI (these peaks were not observed in the raw Illite sample) as show in (Fig. 8). This interpretation was in agreement with Habert et al. (2014) who were proved that the aluminosilicates materials have a high amorphous content, so when placed in an alkaline medium they will hydrolyze and condense, forming new inorganic polymers.

### Adsorption study section

#### Effect of the modification on the surface area (SSA) and adsorption capacity

In this study, the specific surface area of Illite ore and modified Illite (SBMI) after alkaline modification were determined by N_2_ gas adsorption (BET). It was found that SSA for Illite ore was 43 m^2^/g which increased to 104.6 m^2^/g due to the formation of Sodalite which is characterized by high porosity and surface area (Macht et al., 2011). Also, the adsorption capacity for Illite ore was 31.5 mg/g for Ca^+2^, Mg^+2^ which became 94.7 m^2^/g after alkaline modification. The synthetization of amorphous Sodalite material is characterized by sufficient compressive strength and crystalline phase with potential good sorption capacity, and the change of the reaction system chemical compositions (Si/Al and Al/Na molar ratios) affects the nature and quantity of obtained crystalline phases. These results were agreement with Alshameri et al., (2018), Mukherjee et al. (2013a), Karpiński et al. (2015).

#### Effect of SBMI dose on adsorption

The effect of adsorbent dose in batch equilibrium studies is one of the important parameters because it is indicating the sorbent-sorbate equilibrium of a system as well as the adsorbent’s adsorption capacity (Wang et al., 2010).

**Fig. 9:**
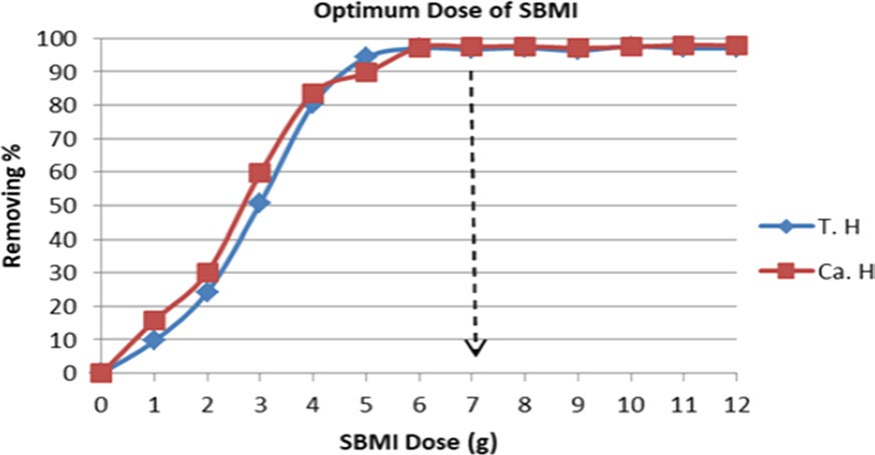
Illustrate the optimum dose of SBMI for completely remove of hardness ions

Therefore, the percentage of removing for undesirable ions was studied using different SBMI dosages and the results are observed in (Fig. 9). It was found that the removal ratio increased rapidly with an increase in the adsorbent dose up to 7 g, and after that, the removal ratio reached almost a constant value. The results indicated that the concentration of total and Ca Hardness ions in the groundwater effluent are reduced by approximately 98%. In the other view, the adsorption efficiency for total, Ca Hardness ions is increased with increasing the adsorbent dose (SBMI). This revealed that the adsorption sites remain unsaturated during the adsorption reaction whereas the number of sites available for adsorption sites increases by increasing the adsorbent dose. This means that the adsorption rate of undesirable ions was directly proportional to the mass of SBMI used, while it is inversely proportional with the increase in the concentration of these ions in the raw water. This can be attributed to the fact that the increase in the removal of unwanted ions is due to the availability of more absorbent surface area (SBMI), or increasing the SBMI dose led to increasing the more active sites thereby, the absorption of undesirable ions accordingly will be increased.

#### Studying the influence of contact time

**Fig. 10:**
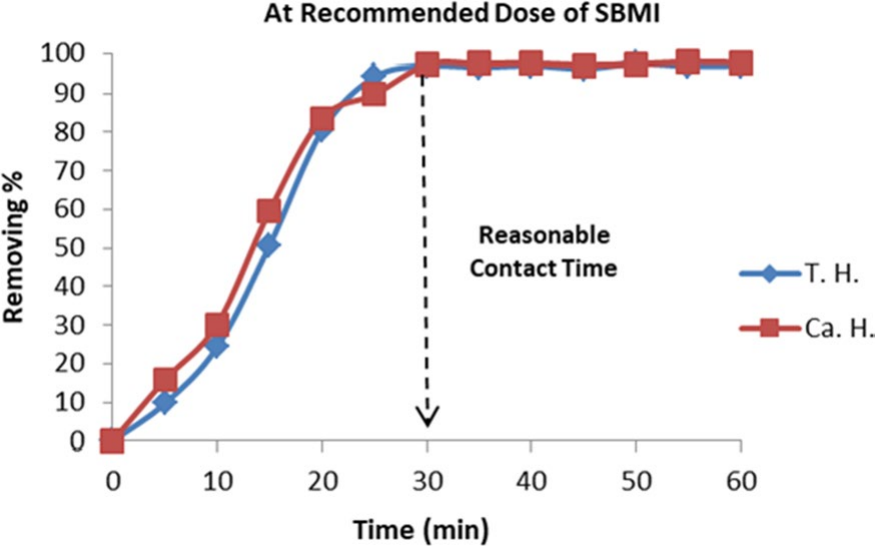
Illustrate the reasonable contact time used for completely remove of hardness ions at recommended dose of SBMI (7 g/l)

The effect of contact time has been studied in raw groundwater contain undesirable cation of total and calcium hardness concentrations**.** This occurred at a fixed dose of SBMI 7 g/L at 25 °C and a natural pH 7.6. The contact time was varied from 0 to 60 min for the concentrations under study and the percentage efficiency was calculated. The results of contact time on adsorption efficiency have been plotted in (Fig. 10). It is found that the optimum contact time for removal of undesirable cations on adsorbent SBMI is 30 min for cations (Mg^+2^, Ca^+2^), these results agree with Chen et al. (2011).

#### Calculation of the optimal alum dose

**Fig. 11:**
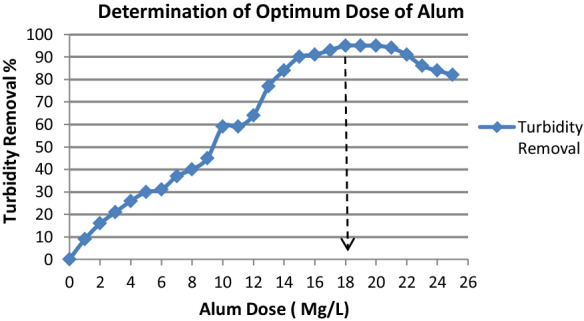
Illustrate the optimum dose of Alum required alum to remove suspended particles of SBMI containing Ca and Mg ions with other impurities is represented by the turbidity

To calculate the optimal alum dose, it had been used the ability of alum to remove suspended particles of SBMI containing Ca and Mg ions with other impurities is represented by the turbidity parameter as shown in (Fig. 11). So With the varying doses of alum were gradually increased, the turbidity removal efficiency improved till the alum dose reached 18 mg/L which known as a recommended dose of alum, at which the highest turbidity removal efficiency was achieved (95%). It had been explained by good flocs, good settling, and low turbidity was formed. After this dose with continuous increasing of the alum doses, the efficacy of turbidity removal began to deteriorate, and the flocs volume became tiny.

#### Study the effect of Illite ore and modified Illite with SBMI in the removing efficiency of hardness ions

**Fig. 12:**
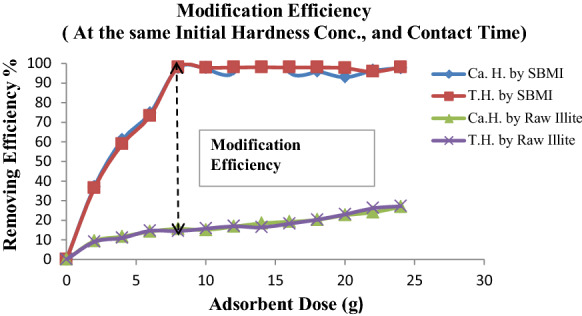
Illustrate the comparison between the Illite ore and modified Illite at different doses of SBMI for removing of hardness ions

It is found that the efficiency removing of hardness ions (Mg^+2^, Ca^+2^) for SBMI is higher than Illite ore as show in (Fig. 12). The study occurred through a series of beakers containing different doses of adsorbents (SBMI, Illite ore) and a constant volume (1000 ml) of El-bora groundwater. This study confirmed the optimum dose of SBMI (7 g/L) which give the highest percentage for removing of hardness ions.

#### Effect of the initial concentration of total and calcium hardness ions on adsorption efficiency of SBMI

To study the ability of SBMI to determine the removing efficiency of hardness salts during the production of water with continuous discharges that mimic the work at the station. Three different groundwater production sites in Assiut governorate are selected with different concentrations of hardness ions with neutral initial pH between 7.4 and 7.8, their chemical analyses are in (Table 2).

**Table 2:** Illustrate the different initial concentration of total and calcium hardness in natural raw groundwater at three different sites

Parameters	T. H	Ca. H	pH
Location			
Permissible limit	*500*	*350*	6.5–8.5
Nagaa Sabaa Village (Low Hardness)	*420*	*270*	7.8
El-Bora Village (Moderate Hardness)	*680*	*580*	7.22
Refa Village (High Hardness)	*1100*	*950*	7.8

**Fig. 13:**
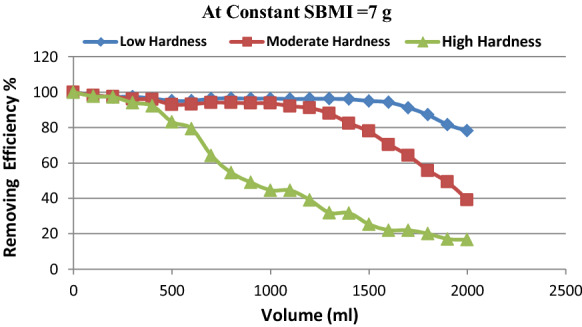
Illustrate the removing efficiency of SBMI at different concentrations of hardness ions in natural groundwater

The results indicated that the highest removal of undesirable cations is recorded when their concentrations at the raw groundwater input were low and moderate hardness (Nagaa Sabaa village, El- Bora Village). On the contrary, the high concentration of groundwater in the Rifa Village has shown a low efficiency in removing these ions, these reverse to the higher driving force for hardness ions to overcome the resistance of mass transport in the liquid phase (Gallab et al., 2017). As a result, the rapid saturation of the binding sites available to hardness causes a decrease in start time with an increased concentration of cations these result are shown in (Fig. 13).

#### The initial pH of raw groundwater

**Fig. 14:**
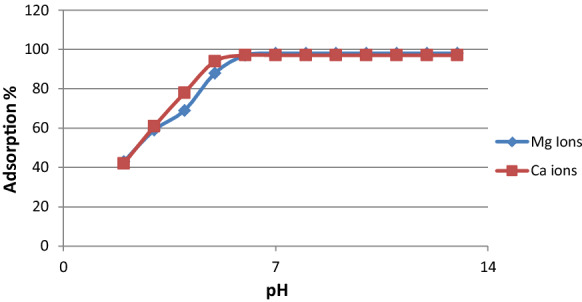
Influence of PH on the adsorption performance of Ca+2, Mg+2 on SBMI

The initial pH of the solution plays an important role in the adsorption process and particularly on the adsorption capacity by controlling the surface charge of the adsorbent and the degree of ionization for the undesirable cations in the groundwater. The increase in the adsorption of (Ca^+2^, Mg^+2^) on SBMI with increasing pH solution can be explained based on the surface charge of SBMI and the metal molecules. The effect of varying pH on the removal percentage of the Mg and Ca ions was investigated and represented in (Fig. 14). The sorption of total Hardness slightly increases at lower pH values until it reaches the range of 6 to 8, at which the adsorption reaches a high level. It may attribute to the sorption process depending on the concentration of the hydrogen ion in the solution. Hydrogen ion strongly competes for the free sites in the structure of the clay leading to a decrease in the percentage of adsorption of ions (Adeyemo et al., 2017). Increasing the pH of solution results in a decrease in H^+^ ion leading to low competition for the vacant exchange sites of the clay, and hence more removal of cations takes place. At pH > 9.5 (high alkaline media), the precipitation of the metal ions hydroxides may take place, thus, the adsorption capacity of SBMI for cations will be still constant (Durães et al., 2018). This was in agreement with which reported by Wang et al., (2021) When the pH of a solution rises above 9.78, the Mg^+2^ in the solution begins to precipitate Mg(OH)_2_, and when the pH rises above 12.78, the Ca^+2^ precipitates Ca(OH)_2_, according to the solubility product rule. As a result, the solution’s ideal pH range is 5.0–9.5. Precipitation is absent inside this range, and just a few protonated functional groups are present**.**

#### Residual alum limit monitoring

**Fig. 15:**
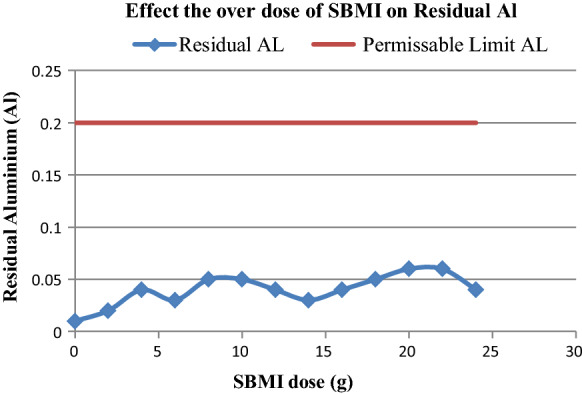
Illustrate the optimum dose of SBMI for completely remove of hardness ions

The World Health Organization (WHO) has determined that the AL residual (Standard Methods 23 RD (3500-AL.B) in drinking water should not exceed 0.2 mg/l. So, the residual of Alum in the output of treated water by modified Illite should be demonstrated. It is affiliated with the aluminosilicates group; thereby, it is mainly composed of aluminum and silica. By studying the residual Alum concentration versus the continuous increase in the modified Illite at the constant of the alum dose (coagulant agent = 100 mg/l), the results appeared that residual Al in treated water did not increase with increasing doses of SBMI, but still under the permissible limit as shown in (Fig. 15). This is consistent with the general characteristics of the aluminosilicates group that does not dissolve in water at the normal temperature as mentioned by Habert et al. (2014), Mahmoud et al., (2021).

## Conclusions

The excess of hardness ions in drinkable groundwater causes many health problems and the conventional treatment techniques for removing them are very high cost and have low production of treated water. Therefore, the present study focused on seeking low-cost and abundant materials using to remove the excess of total and Ca Hardness from drinkable groundwater. The study was conducted by determining the concentrations of (total and Ca Hardness) from all wells in the Assiut governorate and then plotting them concentrations in two different maps of GIS. This paper studied the preparation and modification of Illite ore. It focused on characterizing raw and modified Illite (SBMI) by XRD, SEM, FTIR, and XRF techniques. The application study appeared the ability of the modified Illite for removing Hardness ions from groundwater via two steps (adsorption, and Coagulation-flocculation-sedimentation process), and it was succeeded to remove the Hardness ions (Ca^+2^, Mg^+2^) on ratio reached about 98% for two ions. The present study recommended using this technique, when there is a requirement for large quantities of treated water at a low cost, and it was recommended making a further study on the effect of SBMI as a coagulant agent for removing of algal blooms from drinkable surface water.

## Data Availability

Not applicable.
